# Clinical Lecture on Eclampsia

**Published:** 1900-06-30

**Authors:** Robert Jardine

**Affiliations:** Physician to the Glasgow Maternity Hospital; Examiner in Midwifery to the University of Glasgow; President of the Glasgow Obstetrical and Gynæcological Society.


### Hospital Clinics and Medical Progress.

### CLINICAL LECTURE ON ECLAMPSIA.

By Robert Jardine, M.D., &c., Physician to the Glasgow Maternity Hospital; Examiner in Midwifery to the University of Glasgow; President of the Glasgow Obstetrical and Gynæcological Society.

The case we shall consider to-day is one of eclampsia. The patient is a married primipara aged 21 years. She is the wife of a reservist who is at the front. This is the second case of eclampsia we have had among reservists' wives, and there is no doubt that anxiety has been a predisposing cause in both of them. This patient had had 19 fits before admission. She had been having them for two days, and she cannot remember anything about her illness prior to admission except that she has an indistinct recollection of giving her consent to come in. Otherwise her mind is a complete blank. Swelling of the legs and feet had been noticed some days before her seizure. On admission she was not comatose, but very dull and drowsy. The œdema of the legs was not marked. Her urine was scanty and heavily loaded with albumen. The fundus of the uterus was above the umbilicus. The os was undilated. She had had two hot packs before admission. Her pulse was strong and full.

The treatment adopted was a large dose of salts, a saline injection of two pints (1 drachm each of acetate and chloride of sodium to the pint) under the right breast, and a dose of chloral and bromide. She was also given as much Imperial drink as she could be got to swallow. Next day she was fairly comfortable. No fits had occurred, and she was much brighter. The bowels had moved freely and the kidneys were acting well. I cannot state the amount of urine excreted, as the nurse forgot to measure it, but there was a large quantity. About 24 hours after admission a dead macerated fœtus was expelled by the breech. The labour was practically painless. I have once before seen the same kind of labour with eclampsia. The fœtus must have been dead for some time as it was badly macerated. It has been stated that on the death of the fœtus *in utero* the albumen quickly disappears from the urine. This is the third case I have seen with a macerated fœtus, and in every one of them the urine has been heavily loaded with albumen, so that my experience is directly the opposite of that of some other observers. The patient has made a good recovery. We are still keeping her on milk diet, as there is a faint trace of albumen in the urine.

Puerperal eclampsia is a subject of absorbing interest. So far, many theories of its causation have been advanced, but none of them are satisfactory. It is undoubtedly due to a poison retained in the system, but what the poison is we do not know. It is probably a substance which should have been cleared out of the system through the kidneys. The kidneys are out of gear, so to speak. This is shown by the large amount of albumen usually present, and the very small quantity of urine excreted. The liver is also involved. The poison affects the nervous centres, and causes the convulsions.

The patient usually has some swelling of the limbs and face, and the whole body may be involved. The face shows the swelling most in the morning. Her urine becomes scanty, and in some cases bloody. Severe headache is generally felt for a short time before the convulsions, and she may become very sick. The eyesight is often impaired, and vision may completely fail for a time. In one of my cases the patient was blind for 12 hours during the labour. The actual fit is exactly like an epileptic one, except that the patient does not cry out, neither has she any warning that an attack is coming on. She is apt to bite her tongue. The patient before us had bitten her tongue badly. In such a case blood and frothy mucus comes from the mouth. The patient may injure herself in her struggles. She may remain quite unconscious after the fit, or only be stupid and drowsy for a time. The attacks are apt to recur, and the unconsciousness deepens until she is absolutely comatose. There may be a very large number of fits. In this case 19 occurred. The seizure may be before labour sets in, during labour, or during the puerperium. In my experience the majority are before the onset of labour. I have only seen two cases first seized during the puerperium.

**Treatment.**—Many methods have been tried. In the first place, if albumen is found in the urine of a pregnant woman she should be carefully treated. If the kidneys and bowels are kept acting freely and milk diet taken the chances are that she will escape having fits. Unfortunately, we usually first see the patient when the fits have come on. The first thing to do is to prevent her injuring herself. Place something between her teeth to prevent her biting her tongue. A piece of cork, rubber, or wood, &c., will do. A folded towel drawn between the teeth does admirably, as it will force the tongue back and prevent it being nipped. If we have a poison in the system there are three principal channels through which the system may rid itself of the poison, viz., the bowels, the skin, and the kidneys. To get the bowels to act enemata may be given, but a much more effectual way is by giving large doses of salts—four to six tablespoonfuls in warm water. Croton oil is sometimes used, but it is very uncertain in its action. If the patient cannot swallow, we give the salts through a stomach tube. To get the skin to act a hot air or steam bath, or a hot pack, should be used. To get the kidneys to act, medicines by the mouth are too slow, so we give large saline injections under the breasts or into any parts where the tissues are loose. A drachm of acetate, and the same amount of chloride of sodium are used with each pint of sterilised water. It shoult be used at a temperature of about 104 deg., so as to insure its being at 100 deg. when it enters the tissues. Two or three pints can easily be run under one breast. The skin must be sterilised at the point of puncture. The fluid is quickly absorbed, and the kidneys are flushed by it. If they begin to act well the patient will most likely recover. To keep up the action of the kidneys the patient should be given large quantities of Imperial drink and milk when she is able to swallow. For checking the fits various drugs are used, such as chloroform, chloral and bromide, tincture of veratrum viride, and morphia.

The obstetrical treatment varies according to the condition of the patient. If she is not in labour the uterus should be left alone unless the fits continue, when it may become necessary to deliver. In the present case we left the labour to nature, and the labour was perfectly easy. If the patient is in labour, and the os fully or nearly fully dilated, delivery should be effected. The fits very often cease after the uterus is emptied. Post partum bleeding should be encouraged, as this relieves the system.

The patient may remain comatose for hours, or even days, after delivery, and when she becomes conscious she will have no recollection of what has occurred. She should be kept on milk diet and Imperial drink until the albumen has disappeared from the urine. It usually disappears in a few days. A record of 22 other cases treated by saline infusions will be found in the *British Medical Journal* for May 26th.

